# No need to match: a comment on Bach, Nicholson and Hudson's “Affordance-Matching Hypothesis”

**DOI:** 10.3389/fnhum.2014.00710

**Published:** 2014-09-10

**Authors:** Sebo Uithol, Monica Maranesi

**Affiliations:** ^1^Department of Neuroscience, University of ParmaParma, Italy; ^2^Brain Center for Social and Motor Cognition, Italian Institute of TechnologyParma, Italy

**Keywords:** affordances, mirror neurons, action observation, enactive and extended cognition, social neuroscience

Mirror neurons and canonical neurons are two classes of visuomotor neurons that are activated by different visual stimuli (Rizzolatti and Kalaska, 2012). Mirror neurons respond to a biological effector *interacting* with an object (Gallese et al., 1996), suggesting their role in action recognition, while canonical neurons respond to the presentation of a graspable object (Murata et al., 1997), and are considered crucial in visuomotor transformation for grasping (Jeannerod, 1995).

In their interesting and thought-provoking “affordance-matching hypothesis” Bach et al. (2014) argue that both types of neurons contribute to action understanding. Action hypotheses are posited to be created by means of *object affordances*. Affordances are motor possibilities an object offers (Gibson, 1979). The visual description of an object's intrinsic features are associated with possible motor acts toward that object. A possible neural implementation for this mechanism are canonical neurons. The thus generated action hypothesis based on an object affordance would then be confirmed by the mirror neuron system. When a match between a predicted action (canonical) and an actually observed action (mirror neurons) is confirmed, either the action goal can be predicted based on observed behavior, or behavior can be predicted based on observed goals (see their Figure 1).

We believe, however, that the proposed separation of hypothesis generation and hypothesis matching is not in line with the empirical evidence currently available, and that the division between “interpretation” and “prediction” relies on a cognitivist assumption that is hard to defend. We suggest that enactivist approaches provide a less problematic framework for studying action understanding.

Bach and colleagues are not entirely explicit about the nature of the proposed matching mechanism between affordance and observed action, but we see two options for the proposed division of labor. In the first and admittedly unlikely option, mirror neurons play the role of a quizmaster that knows the answers. If the right hypothesis is posited, all the mirror neuron system has to do is confirm it. In this case, the contribution of the affordances is superfluous, as mirror neurons already extracted all that is needed from the perception of an action, (i.e., the quizmaster knows the answer). Counter evidence for this option exists in the form of mirror neurons that fire in the absence of an affordance to be matched. The auditory mirror neurons reported by Kohler et al. (2002) fire upon the presentation of the sound of an action alone (peanut breaking, paper tearing) without there being an affordance to match, or a prediction to confirm.

But more importantly, virtually all mirror neuron studies (except Bonini et al., 2014a and Caggiano et al., 2009) involved actions performed in the extrapersonal space—out of reach for the monkey. Canonical neurons remain generally silent when an object is in extrapersonal space of the monkey, suggesting a mainly pragmatic (i.e., in terms of possibilities to interact with the object), rather than a metric reference frame (i.e., in terms of physical distance between the object and the observer; Maranesi et al., 2014). This means that the bulk of mirror neuron study reports mirror neuron firing in absence of canonical neuron firing. This, in turn, means that the major part of mirror neuron activity cannot rightfully be framed as “affordance matching,” at least not when canonical neurons are assumed to provide the affordances.

The second and more likely option is that affordance extraction and mirror neuron firing jointly contribute to action understanding by each generating a hypothesis; one based on the object, consisting of one or more actions the object affords, and one about the action the actor is possibly performing (“action classification”; Uithol et al., 2011). When two hypotheses match, they are combined and the action is recognized. However, this means that mirror neuron input is not dependent on the availability of a to-be-matched affordance (i.e., mirror neuron activity is expected without affordances available), which is in line with the empirical evidence as highlighted above, but not predicted by the affordance-matching hypothesis. And also here the fact that canonical neurons fire upon object presentation only in monkey's peripersonal space would mean that canonical neuron-based affordances can only be matched within the monkey's peripersonal space. The only neurons showing canonical properties that could be activated by objects in the extrapersonal space are a recently discovered class of neurons reported by Bonini et al. (2014a). These neurons were dubbed “canonical-mirror neurons” as they show both canonical and mirror properties at the single neuron level. However, the canonical-mirror response to object presentation in the extra-personal space cannot be considered a neural implementation of an affordance, as these neurons do not fire for the same objects in the peripersonal space. Rather, these neurons seem to be involved in an object-triggered action prediction (Bonini et al., 2014a), which is indeed in line with the affordance-matching hypothesis, but emphatically does not generalize to canonical and mirror neurons in general. Additionally, recent findings (Bonini et al., 2014b) revealed that some mirror neurons, besides discharging during action observation, are also active when an action is *not* performed by an actor. This activation can obviously not be interpreted as a match between object affordances and action kinematics, as the latter are absent.

As a solution, one might detach the hypothesis generation and confirmation processes from canonical and mirror neurons; the principle of affordance matching is after all not committed to these classes of neurons. But then we wonder what evidence remains for framing action understanding as “hypothesis generation and testing.” Why is there the need to combine the (in this case two) types of information into a unified representation? We believe that this framing of action understanding as drawing unified and coherent conclusions about observed actions may have been guided by the (cognitivist) assumption that cognition is centered around retrieving information. Alternatively, the framework of enactivism (Varela et al., 1991; Hutto, 2013; Hutto and Myin, 2013) seems to be much more in line with the complexity in action understanding. Enactivism assumes that cognition is not for creating representations about external events, but interacting with the world. In this framework, action understanding can take many guises of which many are best understood as a form of pattern completion: The observer is faced with an incomplete percept of an action, which is then completed based on perceptual mechanisms, mirror mechanisms and even higher associations—e.g., actors-object associations (see Uithol and Paulus, 2013). Importantly, there is no need to combine the different routes into a unified representation of the observed action or inferred action goal. If both object and action information are available, perhaps the classification or prediction process is faster, easier and better, but the current evidence suggest that unifying the types of information into a single match is not necessary.

If action understanding is no longer framed as forming a conclusion about an observed action, but instead in terms of pluriform pattern completion that do not mount (always) to a unified representation, another assumption of the affordance matching hypothesis disappears as well: the difference between interpretation and prediction. Both interpretation (“classification” in our terminology) and prediction involve completing a pattern based on an incomplete percept. This means that the information flow cannot be segmented in “interpretation,” “knowledge,” and “prediction.” Interpretation is not a process upstream of knowledge, and prediction is not a process downstream from it, nor do they represent information flows in opposite directions; both notions refer to the process of sensorimotor action specification.

In all, we believe that the suggestion of the affordance-matching hypothesis that different sources of information can each contribute to action understanding is an important one that could open doors to new lines of research. However, the current evidence does not support the proposed division between hypothesis-generation and hypothesis testing.

## Conflict of interest statement

The authors declare that the research was conducted in the absence of any commercial or financial relationships that could be construed as a potential conflict of interest.

## References

[B1] Bach P. Nicholson T. Hudson M. (2014). The affordance-matching hypothesis: how objects guide action understanding and prediction. Front. Hum. Neurosci. 8:254 10.3389/fnhum.2014.00254 24860468 PMC4026748

[B2] Bonini L. Maranesi M. Livi A. Fogassi L. Rizzolatti G. (2014a). Space-dependent representation of objects and other's action in monkey ventral premotor grasping neurons. J. Neurosci. 34, 4108–4119 10.1523/JNEUROSCI.4187-13.2014 24623789 PMC6705269

[B3] Bonini L. Maranesi M. Livi A. Fogassi L. Rizzolatti G. (2014b). Ventral premotor neurons encoding representations of action during self and others' inaction. Curr. Biol. 24, 1611–1614 10.1016/j.cub.2014.05.047 24998525

[B4] Caggiano V. Fogassi L. Rizzolatti G. Thier P. Casile A. (2009). Mirror neurons differentially encode the peripersonal and extrapersonal space of monkeys. Science 324, 403–406 10.1126/science.1166818 19372433

[B5] Gallese V. Fadiga L. Fogassi L. Rizzolatti G. (1996). Action recognition in the premotor cortex. Brain 119, 593–610 10.1093/brain/119.2.593 8800951

[B6] Gibson J. (1979). The Ecological Approach to Visual Perception. Boston, MA: Houghton Mifflin

[B7] Hutto D. D. (2013). Action understanding: how low can you go? Conscious. Cogn. 22, 1142–1151 10.1016/j.concog.2013.01.002 23375545

[B8] Hutto D. D. Myin E. (2013). Radicalizing Enactivism: Basic Minds Without Content. Cambridge, MA: MIT Press

[B9] Jeannerod M. (1995). Mental imagery in the motor context. Neuropsychologia 33, 1419–1432 10.1016/0028-3932(95)00073-C 8584178

[B10] Kohler E. Keysers C. Umiltà M. A. Fogassi L. Gallese V. Rizzolatti G. (2002). Hearing sounds, understanding actions: action representation in mirror neurons. Science 297, 846–847 10.1126/science.1070311 12161656

[B11] Maranesi M. Bonini L. Fogassi L. (2014). Cortical processing of object affordances for self and others' action. Front. Psychol. 5:538 10.3389/fpsyg.2014.00538 24987381 PMC4060298

[B12] Murata A. Fadiga L. Fogassi L. Gallese V. Raos V. Rizzolatti G. (1997). Object representation in the ventral premotor cortex (Area F5) of the monkey. J. Neurophysiol. 78, 2226–2230 9325390 10.1152/jn.1997.78.4.2226

[B13] Rizzolatti G. Kalaska J. (2012). Voluntary movement: the parietal and premotor cortex, in Principles of Neural Science, 5th Edn., eds Kandel E. Schwartz J. Jessell T. Siegelbaum S. Hudspeth A. J. (New York, NY: McGraw-Hill), 865–893

[B14] Uithol S. Paulus M. (2013). What do infants understand of others' action? A theoretical account of early social cognition. Psychol. Res. 78, 609–622 10.1007/s00426-013-0519-3 24100453

[B15] Uithol S. van Rooij I. Bekkering H. Haselager W. F. G. (2011). Understanding motor resonance. Soc. Neurosci. 6, 388–397 10.1080/17470919.2011.559129 21391148

[B16] Varela F. J. Thompson E. Rosch E. (1991). The Embodied Mind: Cognitive Science and Human Experience. Cambridge, MA: MIT Press

